# Quantitative Study of Spodumene by Time-of-Flight Secondary Ion Mass Spectrometry (tof-SIMS)

**DOI:** 10.3390/molecules30071552

**Published:** 2025-03-31

**Authors:** Xijuan Tan

**Affiliations:** Laboratory of Mineralization and Dynamics, College of Earth Sciences and Land Resources, Chang’an University, 126 Yanta Road, Xi’an 710054, China; tanxijuan@hotmail.com or tanxijuan@chd.edu.cn

**Keywords:** spodumene quantification, matrix-matched standard calibration, tof-SIMS, ionization efficiency

## Abstract

In this paper, the quantitative feasibility of time-of-flight secondary ion mass spectrometry (tof-SIMS) for major and minor elements in spodumene was evaluated in terms of calibration method with a matrix-matched or non-matrix-matched standard and an internal standard element using Al or Si. The matrix-matched standard calibration method was studied using spodumene 503R as the external standard and unknown sample, with signal intensities collected under positive ion mode using 100 µm of raster size. The sensitivities of Li, Na, Al, Si, Mn, and Fe were obtained by applying the sample-standard bracketing method, and the corresponding concentrations were given as the division of signal intensities by sensitivities. It was found that there were no significant differences between concentration results using Al and Si as the internal standard element. After 100% normalization, the concentrations at a 95% confidence level of matrix Li_2_O, Al_2_O_3_, and SiO_2_ in oxide form were found to be 7.62 ± 0.27%, 27.68 ± 0.10%, and 64.32 ± 0.29%, respectively, which agreed with those from LA-ICPMS measurements and/or EPMA analyses. The comparison of the minor components including Na_2_O, MnO, and FeO showed that the contents from tof-SIMS were consistent with references from LA-ICPMS, giving ratios within 0.98–1.02. Furthermore, the element behavior investigation of NIST SRM 610 showed that the ionization efficiencies differentiated among the studied elements, resulting in far lower sensitivities of Li, Na, Mn, and Fe in spodumene than the values from the proposed matrix-matched standard calibration method. Thus, the matrix-matched standard calibration method for element determination of spodumene by tof-SIMS was recommended. The successful determination of major and minor elements in spodumene also promises the future application of tof-SIMS to trace element quantification.

## 1. Introduction

The in situ quantitative analysis at micro- and nanoscale of geological samples with high precision and accuracy has become an important strategy in the study of magmatic, metamorphic, and sedimentary processes in the last several decades [1,2]. Currently, the reported in situ techniques for element determination in geochemistry mainly include electron probe microanalyzer (EPMA) [3], laser ablation inductively coupled plasma mass spectrometry (LA-ICPMS) [4,5], secondary ion mass spectrometry with the mass analyzer of sector field (sf-SIMS) [4,6], and sensitive high resolution ion microprobe (SHRIMP) [7].

Time-of-flight SIMS (tof-SIMS) is a static SIMS in which an ion dose is less than 10^13^ ion/cm^2^ and the secondary ions are measured by their time of flight from the sample surface to the detector [8]. Such an instrument is chemically specific and enables the quasi-simultaneous detection of all secondary ions during a single measurement, allowing for sensitive analyses of a sample with minimum damage due to the usage of a pulsed primary ion beam [9]. The tof-SIMS analyses are also characterized by a wide mass range, high mass resolution, and high spatial resolution up to 50 nm [10]. Furthermore, the sample mass required for one assay cycle is only a few picograms about 10^−1^ to 10^−5^ of the sample consumption in SHRIMP or sf-SIMS analysis [11]. These specific properties make tof-SIMS highly suitable for in situ element analysis of small and/or rare samples in various geological studies [12,13,14]. For example, by using tof-SIMS rather than other analytical techniques such as EPMA, sf-SIMS, and LA-ICPMS, Saunders et al. [15] identified zoning of Li on a spatial scale of ca. 5–10 µm in orthopyroxene crystals from the May 1982 eruption of the Mount St. Helens volcano, U.S.A., which assured the successful investigation of the associated pre-eruptive magmatic processes. This surface analytical tool was also applied to characterize the micro-morphological and compositional features of five rock chip samples from CE-5 lunar soil [16]. Based on the obtained element/molecular ion distribution images, the authors identified the minerals involved in the rock chip samples along with their occurrence states and distribution. Because of the excellent lateral resolution, they successfully probed uncommon vesicle-like patterns of the minerals which may not be captured by conventional electron microscopy. Collectively, tof-SIMS has been extensively utilized in element imaging study and chemical composition identification in terrestrial and extraterrestrial samples.

According to the literature, there were several reports on the application of tof-SIMS to element quantification of geological samples by introducing the relative sensitivity factor (RSF) method. Marques et al. [17] measured selected major and trace elements (e.g., Ma, Na, K, V, La, and Ce) in metal inclusions from rock samples. But they highlighted that the relative quantification of elements will depend on mass interferences and the quality of the standards used. In other words, despite there being similar compositions between the studied melt inclusions and the external calibration standard, mass interferences and low counts made it difficult to quantify some elements, such as Fe, Ni, Co, Cu, and Zn. Long et al. [11] constructed a new tof-SIMS producing an O_2_^−^ beam of ca. 5 µm with a beam intensity of ca. 5 nA and a mass resolution of >20,000 (FWHM), with determination accuracy of rare earth elements and Ti in zircon samples investigated. The element quantification via this newly designed tof-SIMS was done based on the matrix-matched standard calibration method, for which an extra reference mass peak must be used. Furthermore, such a reference peak should meet the requirements of having a comparable mass number and spectral intensity with those of the targeted elements. For example, ^150^(ZrSiO_2_) and ^28^SiO_2_ were chosen as the reference peaks for rare earth elements and ^48^TiO, respectively. Recently, Wang et al. [18] assessed the quantitative accuracy of trace elements (e.g., Sr, Mn, and ^54^Fe) and halogens (e.g., F and Cl) in the apatite by tof-SIMS using the matrix-matched standard calibration method, showing quantification precisions of 4.41–11.68% and relative errors of 0.07–10.92%. The authors also reported that apatite quantification calibrated by the non-matrix-matched standard NIST SRM 610 can be achieved using O as the matrix element in the positive ion mode, with ratios of true values to the measured within 3.

Spodumene is an aluminosilicate of Li with a general chemical formula of LiAl(SiO_3_)_2_ [19]. This Li-bearing mineral, which occurs frequently in pegmatite Li deposits, is of substantial economic value for Li extraction at an industrial scale [20]. In the case of geochemistry, the chemical compositions of spodumene samples are regional characteristics [21], and the element concentration levels are valuable fingerprints to trace Li mineralization stages of spodumene pegmatites [22]. It is no doubt that accurate element determination of spodumene is a prerequisite for such geological applications. The literature showed that EPMA was frequently used for spodumene quantification, but information on Li cannot be obtained directly by this in situ method [23]. LA-ICPMS is another in situ approach for the quantification of major and trace elements in spodumene with good precision and high accuracy [21]. To the best of our knowledge, however, there has been no study on the quantification of spodumene by using tof-SIMS.

In this current work, we exploited the feasibility of tof-SIMS in the determination of major and minor elements in spodumene for the first time. The quantifications of element compositions in spodumene from tof-SIMS analysis with data calibrated by the matrix-matched standard and non-matrix-matched standard were compared. Furthermore, the effect of the internal standard element on quantification accuracy and the stability of single intensity after surface precleaning using gas gun sputtering were studied in detail. Finally, this proposed tof-SIMS quantification strategy was also evaluated by the analytical accuracy of spodumene 650, which has known chemical compositions [21].

## 2. Results and Discussion

### 2.1. Quantification Theory of tof-SIMS

Since the secondary ion emissions of the sample are recorded in intensity units, the signal intensity is theoretically proportional to concentration. However, it is a fact that all secondary ion emissions exhibit a strong sensitivity to the chemistry of the sample surface during sputtering, which is known as the matrix effect. This causes secondary ion yield variations that span orders of magnitude, making the direct conversion of secondary ion intensities into element concentration complicated. Such conversions thus require the removal of the matrix effect. By using a matrix-matched reference material with the standard and sample analyzed under identical conditions, an RSF method had been recommended for the quantification application of dynamic SIMS [24,25]. But in the case of tof-SIMS, it was reported that the quantification can be eased by reduced matrix variations when the studied element of interest exists in submonolayer coverage on some well-defined support [24]. Here, we introduced the sensitivity (*S*), the counts per unit of concentration, which was reported by Longerich et al. in element quantification by LA-ICPMS [26], to connect element concentration of the sample and the corresponding signal intensity from tof-SIMS analysis for the first time (Equation (1)):

(1) $$C_{x}^{sam}=\frac{I_{x}^{sam}}{S_{x}}$$

where $I_{x}^{sam}$ is the signal intensity of element *x* from tof-SIMS analysis, $C_{x}^{sam}$ is the concentration of element *x* in the studied sample, and *S*_x_ is the normalized sensitivity of the tof-SIMS for the studied element. The normalized sensitivity can be determined on a matrix-matched standard material which is analyzed under the same conditions as the sample. With the analytical bias from the concentration difference between the standard material and the sample being corrected by an internal standard element, the normalized sensitivity of element *x* in the sample is given as follows (Equation (2)):

(2) $$S_{x}=\frac{I_{x}^{\mathrm{std}}}{C_{x}^{s\mathrm{td}}}\frac{I_{IS}^{s\mathrm{am}}}{C_{IS}^{s\mathrm{am}}}\frac{C_{IS}^{s\mathrm{td}}}{I_{IS}^{s\mathrm{td}}}$$

where $I_{x}^{std}$ is the signal intensity of element *x* in the standard material; $C_{x}^{sam}$ is the concentration of element *x* in the standard material; $I_{IS}^{sam}$ is the signal intensity of the internal standard element (*IS*) in the sample; $C_{IS}^{sam}$ is the concentration of the internal standard element in the sample; $I_{IS}^{std}$ is the signal intensity of the internal standard element in the standard material; and $C_{IS}^{std}$ is the concentration of the internal standard element in the standard material.

### 2.2. Element Determination of Spodumene by tof-SIMS Using the Matrix-Matched Standard

To evaluate the feasibility of tof-SIMS for spodumene quantification, spodumene 503R, with concentration values from LA-ICPMS analysis as a reference (see below section), was used as both the calibration standard material and the unknown sample in this study. Since the secondary ions produced by ion bombardment can be positive and/or negative [27], the element ionization behavior of spodumene by using a Bi^+^ primary ion beam was studied before quantification analysis. The mass spectra of spodumene collected under the positive mode and negative mode are given in Figure S1. Compared to the results obtained under the positive ion mode (see Figure S1a), it was found that the signal intensities of the targeted elements Li, Na, Al, Si, Mn, and Fe were extremely low and cannot be observed directly when applying the negative ion mode (see Figure S1b). Additionally, the extremely low signal intensities within a mass range from 55 to 115 amu under the negative ion mode (see Figure S1c) clearly showed that the probability of oxide formation of the studied elements was low. This demonstrated that the positive ion yielding efficiencies of the studied elements in spodumene were prominently higher than the efficiencies of negative ions.

From the above discussion, the mass spectra of spodumene were then collected under the positive ion mode in the subsequent work. The signal intensities of elements Li, Na, Al, Si, Mn, and Fe were exported after mass spectra calibration. Generally, the nominal mass of each isotope was set as 1.000 which binned from 0.300 to 1.300, and the signal intensity for each mass (*m*) was thus the total value from *m* − 0.7 to *m* + 0.3. With the matrix Al or Si as the internal standard element, the sensitivities of the studied elements were then obtained by using Equation. 2, and the corresponding results were summarized in Table 1.

As shown in Table 1, the sensitivities were found to be element dependent for spodumene, with values following a sequence of Mn > Na > Fe > Li > Al > Si in these current data acquisition conditions. It was also found that the sensitivity values using Al as the internal standard element were comparable with those using Si as the internal standard element. With the obtained sensitivities, the element concentrations of spodumene 503R were calculated using Equation 1, and the results in oxide form (wt %) are given in Table 2. Here, in order to be consistent with the typical data expression of geological samples in EPMA and LA-ICPMS analysis, the concentration results of elements Mn and Fe from tof-SIMS measurements were given in the oxide form of MnO and FeO, respectively, even though the valence of the two elements cannot be distinguished by these three techniques. The quantification results in Table 2 show that the concentrations using Al as the internal standard element were comparable with those applying Si as the internal standard element. For example, the contents of Li_2_O (2σ) were 7.59 ± 0.24% and 7.57 ± 0.27% by using Al and Si as the respective internal standard element. It can thus be deduced that both the matrix Al and Si were plausible to be the internal standard element for spodumene quantification by tof-SIMS. Additionally, the yielded relative standard deviations (RSDs, n = 6) of spodumene determination were less than 4.8%, showing the good precision of this developed tof-SIMS method for element quantification of spodumene. In this work, the obtained concentrations of all the studied elements further underwent 100% normalization, and the normalized concentrations are collected in Table 3. It is clear that the concentrations of matrix Li_2_O, Al_2_O_3,_ and SiO_2_ were in a range of 7.02–7.97%, 27.53–27.87%, and 63.72–64.86%, respectively. It can also be seen that the average concentrations of minor components Na_2_O, MnO, and FeO were 0.089% (±0.003%, 2σ), 0.058% (±0.002%, 2σ), and 0.24% (±0.007%, 2σ), respectively.

### 2.3. Comparison of tof-SIMS to LA-ICPMS and EPMA for Spodumene Quantification

To evaluate the quantification accuracy of this tof-SIMS method, the results were compared to the concentrations obtained from LA-ICPMS and EPMA analysis. As can be seen in Table 3, the concentrations of major components Li_2_O, Al_2_O_3,_ and SiO_2_ in spodumene 503R from tof-SIMS were consistent with the results from LA-ICPMS analyses, giving ratios in a range of 0.95–1.01. It was also found that the contents of the minor components Na_2_O, MnO, and FeO from the two methods were in high agreement, yielding concentration ratios of 0.98–1.02. Furthermore, the comparison of tof-SIMS and EPMA in the case of spodumene determination showed that the concentrations of matrix Al_2_O_3_ and SiO_2_ were comparable. This demonstrated the capability of tof-SIMS for element quantification of spodumene samples. However, it is worth noting that there were relatively large concentration deviations of minor elements, giving ratios higher than 1.2. Considering that spodumene 503R is a natural mineral, a backscattering electron (BSE) imaging study was then carried out to check the homogeneity of this mega crystal. The BSE image (see Figure S2) showed that there existed other mineral inclusions on the surface of this spodumene 503R. It can thus be deduced that such obvious concentration differences of the minor elements should be ascribed to the heterogeneous distribution of minor elements Na, Mn, and Fe in the studied sample and the significant measurement errors from the relatively small diameter of electron beam (i.e., 1 µm) used in EPMA analysis. Apparently, to obtain the bulk information of the minor elements Na, Mn, and Fe, a representative sampling mass of this spodumene sample can be achieved by using 100 µm raster size in tof-SIMS analysis, which was equivalent to the usage of 40 µm spot size in LA-ICPMS analysis. Collectively, spodumene samples can be accurately determined by tof-SIMS based on the matrix-matched standard calibration method.

### 2.4. Element Behavior Study of the Non-Matrix-Matched Standard NIST SRM 610

In this work, the viability of the non-matrix-matched standard calibration method for spodumene 503R quantification by tof-SIMS was also assessed. Since NIST SRM 610 has been extensively utilized as an external standard in element quantification of geological samples by in situ analytical techniques [21,28,29], the element behavior of NIST SRM 610 in tof-SIMS analysis was investigated. Results showed that the signal intensities of elements Li, Mn, and Fe in this standard, with concentrations of 468 ± 24, 444 ± 13, and 458 ± 9 µg/g, respectively [28], were less than 1000 cts, and in particular the intensity value of Li was lower than 50 cts. It was also found that the signal intensity of Na in NIST SRM 610 was only 26.4% of that in spodumene 503R, but the corresponding concentrations of Na_2_O were 13.4 ± 0.3% and 0.091 ± 0.009%, respectively. This might be caused by the surface contamination, or the poor secondary ion yields of NIST SRM 610 under the current bombarding condition by the Bi+ primary ion.

**Figure 1:**
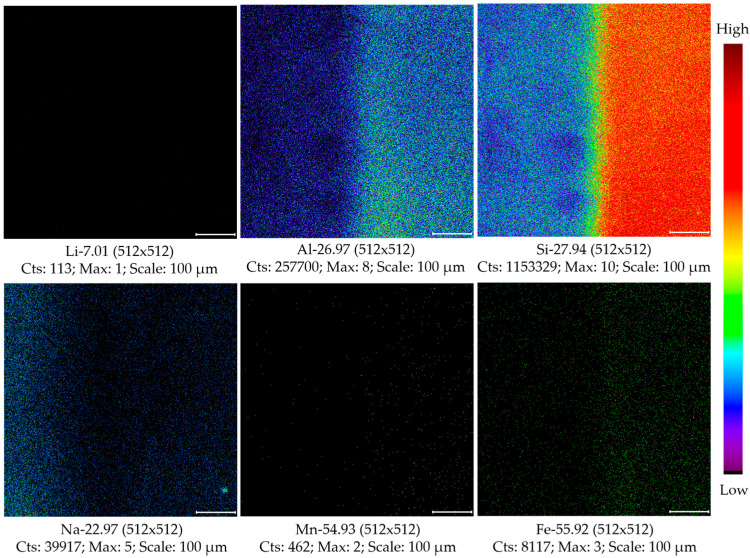
Images of the studied elements in NIST SRM 610 with and without gas sputtering. Here, the image was studied using a raster size of 600 µm, with left-half part and right-half part corresponding to unsputtered sample surface and sputtered sample surface. The utilized argon gas ion gun with a source beam energy of 3 kV had a beam current of 150 nA.

To further evaluate the potential effect of any surface contaminants on element signal intensities, the surface of NIST SRM 610 with an area of 600 × 600 µm^2^ was sputtered using an argon ion gas gun for 0, 20, 40, and 60 s. The mass spectra after each sputtering were collected in the center of the sputtered region of 100 × 100 µm^2^ (see Figure S3). It was found that there were no significant differences between the signal intensities of Li and Mn with and without gas ion sputtering. But the signal intensities of Al, Si, and Fe increased in different degrees with 20 s of gas ion sputtering, and the corresponding increments were found to be irrespective of sputtering time. Here, the signal intensity of Si in NIST SRM 610 was observed to be 1.08-fold to that of Si in spodumene 503R, which agreed with the corresponding concentration ratio of the two (i.e., 69.7 ± 0.5% of SiO_2_ in NIST SRM 610, and 63.90 ± 0.16% of SiO_2_ in spodumene 503R). It was also found that the signal intensity of Na decreased prominently, and the intensity decrement reached about 70% after the sputtering. Such element behavior of NIST SRM 610 can also be observed from the imaging study of this standard with and without sputtering treatment (see Figure 1), which demonstrated that the extremely poor ionization efficiencies of the studied elements except Si and Al in this glass silicate standard should not be due to surface contaminations but rather the element ionization behaviors under the bombardment using the primary ion beam of Bi^+^. In a previous study of Li isotope by sf-SIMS using a primary beam of O^−^ ions, it was reported by Denny et al. [30] that the fused-rock SRMs (e.g., USGS SRM BCR-2G) ionized Li 2–3× more efficiently than synthetic glass SRMs (e.g., NIST SRM 612), and the authors ascribed this to the differences in major oxide chemistry and the reduced nature of the synthetic glasses relative to their fused counterparts. Ray and Hart [31] suggested that glass standards cannot be used for trace element analysis and there was need for caution in using glass standards when measuring crystalline samples by sf-SIMS due to the fact that elements were preferentially sputtered from crystals. Andersen and Hinthorne [32] also showed that the yields of sputtered ions were greatly affected by the surface chemistry of the sample. Hence, the distinctly low secondary ion yields of element Li, Na, Mn, and Fe in NIST SRM 610 might be attributed to the less crystalline nature of this synthetic soda-lime silicate standard.

By calibrating against NIST SRM 610, the further sensitivity study of spodumene 503R using Si as the internal standard element showed that the normalized sensitivities of Li, Mn, and Fe were lower than 10. Clearly, the yielded sensitivities using this non-matrix -matched standard were far less than the values obtained by the proposed matrix-matched standard calibration method. This revealed the incapability of this synthetic soda-lime silicate standard NIST SRM 610 as an external standard for spodumene quantification by tof-SIMS.

### 2.5. Effect Study of Surface Sputtering on Spodumene Analysis

Sputtering the sample surface using a gas ion gun is a plausible way to preclude the possible influence of undesired contaminants during tof-SIMS analysis. In this work, the sputtering effect from the argon gas ion gun on spodumene analysis was investigated. With the sample surface of 600 × 600 µm^2^ sputtered, the mass spectra were collected immediately using a raster size of 100 µm. Results for 0, 20, 40, and 60 s of sputtering are shown in Figure S4. It was found that the signal intensities of Li, Na, Al, and Si increased obviously after sputtering. However, the increments were irrespective of sputtering time, yielding the intensity ratios of around 3.8, 2.9, 3.2, and 7.9, respectively. It was also found that there were no significant variations of the signal intensities of Fe, while the signal intensity of Mn decreased dramatically and exhibited about 90% of intensity loss after sputtering. Yet the reason for such significant intensity loss of Mn in spodumene remained unclear. The significantly different element behaviors of spodumene and NIST SRM 610 under the argon gas sputtering environment reconfirmed the matrix-matched standard calibration method was preferred for spodumene quantification.

**Figure 2:**
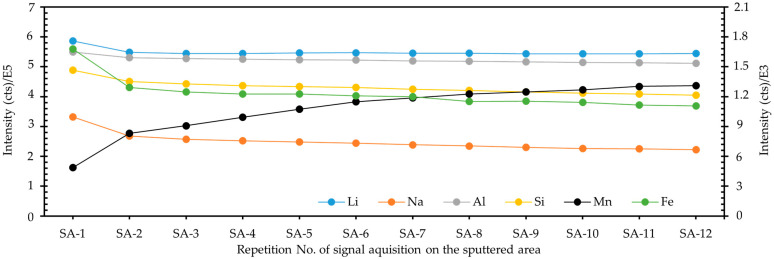
Profiles of signal intensities of elements with running time after argon gas sputtering. Here, the sputtering time before mass spectra collection was 60 s. The utilized argon gas ion gun with a source beam energy of 3 kV had a beam current of 150 nA. An area of 600 × 600 µm^2^ was sputtered by the gas ion gun, and the signal acquisition (SA) by Bi+ ion beam was done in the center of the sputtered region with a raster size of 100 µm.

In this work, to study the long-term effect of gas sputtering on spodumene quantification, 12 consecutive signal acquisitions of mass spectra at the same position were then conducted after 60 s of sputtering. The signal intensities of the studied elements are graphically shown in Figure 2, with Mn and Fe presented as the secondary axis. It can be seen from Figure 2 that the signal intensities of Li, Na, Al, Si, and Fe decreased gradually with running time after the stop of sputtering, while the element Mn exhibited an opposite trend in signal intensity. 

**Figure 3:**
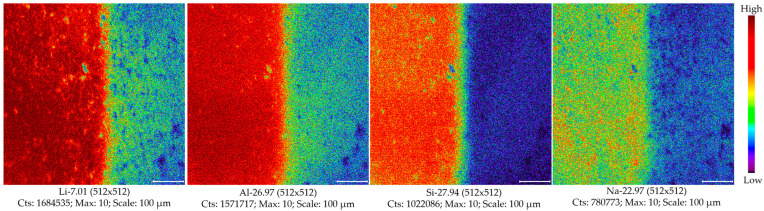
Element images of spodumene 503R with and without gas sputtering. Here, the image was taken using a raster size of 600 µm. For each element image, the left-half part was the sputtered sample surface with 60 s of argon gas ion sputtering, while the right-half part was the unsputtered sample surface. The utilized argon gas ion gun with a source beam energy of 3 kV had a beam current of 150 nA.

It was worth noting from the obtained element images of Li, Al, Si, and Na that the surface topography of this mineral was obviously modified by gas sputtering (see Figure 3). However, the changes in the sample surface were observed to be element-dependent. It is thus clear that the surface chemistry of spodumene was affected by argon gas ion sputtering. Collectively, such changing trends in the element signal intensities might be most likely due to the destruction of the oxidized phase known to exist on the surface by argon ion sputtering [32]. Additionally, it was found that the intensity variations between the first two collections were more prominent than the subsequent acquisitions. To exclude the possible influence from argon ion gas sputtering and analyze spodumene on the less affected and more representative sub-surface, it is highly recommended to preclean the sputtered area using a Bi^+^ ion beam for at least 2 min.

### 2.6. Application of the Proposed tof-SIMS Quantification Method for Spodumene Analysis

In this work, the developed tof-SIMS quantification strategy was also applied to the determination of spodumene 650, which was from the Kaluan deposit in Xinjiang, China [21]. The element concentrations and the corresponding 100% normalized results of spodumene 650 by tof-SIMS are summarized in Table S1 and Table 4.

From Table S1, it was found that there were no significant differences in the obtained contents calibrated against with Al and Si, which reconfirmed the viability of both elements as the internal standard in spodumene quantification. Furthermore, it is clear from Table 4 that the 100% normalized contents of Li_2_O, Al_2_O_3_, SiO_2_, Na_2_O, MnO, and FeO were 7.84 ± 0.15%, 27.22 ± 0.003%, 64.65 ± 0.34%, 0.115 ± 0.457%, 0.149 ± 0.002%, and 0.020 ± 0.001%, respectively. Apparently, the concentration results were consistent with the references from LA-ICPMS and/or EPMA analysis. The comparable results further revealed the feasibility of this tof-SIMS based on matrix-matched standard calibration in element quantification of spodumene, giving the quantification limits lower than 60, 40, and 10 µg/g for minor trace elements Na, Mn, and Fe, respectively.

## 3. Materials and Methods

### 3.1. Spodumene Sample and Silicate Glass Standard Material

The spodumene (503R) utilized in this work was sampled from the No. 503 pegmatite vein of the Dahongliutan Li deposit in Xinjiang, China. This sample was a megacrystic in hand specimen with a crystal size of 4 cm in length and 1 cm in width. The spodumene 503R was prepared in the form of thin section. In brief, the sample was cut to a thickness of about 50 µm and carefully mounted on a glass bead. The thin section was then polished until a flat surface of the sample was obtained. Thereafter, the sample bead was cleaned using ethanol (99.7%) and subsequently ultrapure water (Milli-QR EQ 7000, Millipore, Bedford, MA, USA) in a sonication bath.

The reference glass NIST SRM 610, which has been frequently used in LA-ICPMS analyses for concentration calibration of geological samples, was taken as the non-matrix-matched calibration standard in this study. This standard material was produced by the National Institute of Standards and Technology of USA (Gaithersburg, MD, USA) by melting high-purity quartz sand, alumina, soda ash, and calcium carbonate with up to 61 trace elements of 500 µg/g nominal concentration added. The preferred concentration values used as reference for this silicate standard were from the GeoReM database [28].

### 3.2. tof-SIMS Apparatus

The study of spodumene quantification by tof-SIMS was carried out on a PHI nano TOF 3 tof-SIMS (ULVAC-PHI, Inc., Chigasaki, Kanagawa, Japan) located at the Laboratory of Mineralization and Dynamics, Chang’an University. This tof-SIMS, which enables a flight path of 2 m for the secondary ions, is equipped with a Bi cluster liquid metal ion gun, a double mode neutralization system (i.e., the electron flood gun and the argon ion gun), a TRIFT analyzer, and an MCP detector. Here, note that the argon ion gun can be switched to a sputtering phase and then used as the gas gun to sputter the sample surface when precleaning is necessary. All the mass spectra were given by software TOF-DR 3.0.

The Bi cluster liquid metal ion gun was used to produce the primary ion beam, such as Bi^+^ and Bi_3_^++^. Considering the element compositions of spodumene, the Bi^+^ ion beam was chosen as the primary ion beam for spodumene detection to obtain a relatively higher yield of atomic ions. After the optimization of beam steering, around 5 nA of the current of Bi^+^ ion beam at 30 kV using 200 µm of aperture size was obtained. Before data acquisition, mass calibration was carried out to obtain high mass scale accuracy. In brief, the ratios of mass deviation to three masses from low to high were controlled less than 0.01. Generally, the Ion package of CH_3_ (15.0235 amu), C_2_H_3_ (27.0235 amu), and C_3_H_5_ (41.0391 amu) was selected for mass calibration under the positive ion mode, while the ions including CH (13.0078 amu), C_2_H (25.0078 amu), and C_4_H (49.0078 amu) were calibrated under the negative ion mode. With spectral calibration done, a mass resolution (m/∆m) of 12, 000 can be achieved. During quantitative analysis, the high mass resolution mode of the Bi^+^ ion beam with a pulse width of 2 ns, which was also known as the spectra or bunched mode, was chosen for the quantification study. Here, a raster size of 100 µm yielding an analytical area of 100 × 100 µm^2^ was used.

In this work, charge neutralization was done by adding a reasonable sample bias voltage when the low energy electron flood gun was on. The sample bias voltage optimization was carried out with the detector in “off” mode, and the optimal value was given when the signal spot on the fluorescent plate was in the quasi-static status. The values of sample bias voltage for spodumene 503R and NIST SRM 610 were 3337 v and 3647 v, respectively.

### 3.3. Data Acquisition and Processing

In this work, the analyses were done using the standard-sample bracketing (SSB) method. Here, for the matrix-matched standard calibration method, the spodumene 503R was used as both the standard and unknown sample. All data acquisition on spodumene 503R were carried out on surface regions without inclusions under optical observation. Generally, one complete assay cycle consisted of a sequence of 2 analyses of the standard (503R or NIST SRM 610), 6 analyses of spodumene samples, and 2 analyses of the standard (503R or NIST SRM 610).

All data were collected using 512 × 512 pixels of scanning pixel and a limited acquisition mode of 2 frames, which corresponded to a data collection time of 1.06 min and an ion dose of 1.47 × 10^11^ ion/cm^2^. Here, spodumene 503R or NIST SRM 610 was defined as the external standard. The integral signal intensities of Li (7.0161 amu), Na (22.9895 amu), Al (26.9799 amu), Si (27.9751 amu), Mn (54.9360 amu), and Fe (55.9337 amu) in spodumene, with corresponding mass resolutions of 2712.69, 3645.43, 2958.96, 2460.73, 6154.06, and 6401.07, were outputted from the mass spectra, and the quantification was done using Al or Si as the internal standard element. The obtained element concentrations were normalized to 100% and expressed as 100 wt % oxide.

### 3.4. LA-ICPMS Analysis

A single quadrupole ICPMS (Agilent 7900 ICP-MS, Santa Clara, CA, USA) connected to a 193 nm ArF excimer Analyte Excite LA system (Photon Machines, Belgrade, MT, USA) was used to measure the major and minor elements of spodumene as the reference. The detailed descriptions of the ICPMS and LA system were given in our previous work [33,34]. Here, the sample aerosol from the ablation cell was carried out by helium (99.999% purity) and then mixed with the make-up gas argon (99.996% purity) by using a T-connector before entering the ICP. After daily optimization using NIST SRM 610, a ratio of ThO^+^/Th^+^ for oxide formation lower than 0.5% and a signal intensity ratio of ^238^U^+^/^232^Th^+^ near 1.05 were achieved.

Using NIST SRM 610 as the external calibration standard, the quantification of spodumene 503R by LA-ICPMS followed the method reported by Tan et al. [21]. In brief, after 2 spot analyses of NIST SRM 610, 10 spot analyses of spodumene samples were conducted, and then another 2 spot analyses of NIST SRM 610 followed. The utilized spot size, laser repetition, laser energy fluence, helium gas flow rate, and argon gas flow rate were 40 μm, 7 Hz, 3.91 J/cm^2^, 0.8 L/min, and 0.7 L/min, respectively. Each analysis consisted of a 15 s background signal acquisition and 40 s data collection, with data collected in time resolved mode. The data reduction was carried out using “Stalquant” [35]. Quantification was done with Al as the internal standard element or via matrix normalization (100 wt % oxides) [36], with element concentration results shown in 100 wt % oxide.

### 3.5. EPMA Analysis

EPMA analysis of spodumene 503R was done on a JEOL JXA-iHP200F instrument (Jeol Ltd., Akishima, Tokyo, Japan). The measurement was carried out using an acceleration voltage of 15.0 kV, a probe current of 20 nA and an electron beam diameter of 1 µm. The acquisition times were 20 s on peak and 10 s on each background position. All the studied elements were measured using *K*_α_ lines. Here, element Na was measured on TAP, elements Si and Al were measured on TAP (L type), and elements Fe and Mn were determined on LIF. Natural minerals wollastonite, albite, corundum, fayalite, and pyrophanite were used as standards for Si, Na, Al, Fe, and Mn, respectively. Data were collected at 10 different points of the samples, and element concentrations were calculated by using the CitZAF interelement correction model [37]. The results were expressed as oxide form in wt %.

## 4. Conclusions

In this work, we introduced the normalized sensitivity to explore the capability of tof-SIMS for spodumene quantification. By using the matrix-matched standard calibration method, the major and minor elements including Li, Na, Al, Si, Mn, and Fe in spodumene were successfully determined. The 100% normalized concentrations were found to highly agree with those from LA-ICPMS measurements, giving content ratios within 0.95–1.02. When it comes to the non-matrix-matched standard calibration method, it was found that the ionization efficiencies of the studied elements in NIST SRM 610 glass silicate standard differentiated significantly, yielding far lower normalized sensitivities of Li, Na, Mn, and Fe in spodumene than the values from the developed matrix-matched standard calibration method. The effect study of gas sputtering showed that there were distinct differences of element behaviors between spodumene and NIST SRM 610, reconfirming that NIST SRM 610 was unfavored for spodumene quantification by tof-SIMS. Additionally, cleaning the sputtered area using the Bi^+^ ion beam at least 2 min before quantification was necessary due to the gas sputtering effect on the surface topography and chemical properties of spodumene. This study demonstrated that tof-SIMS can be applied to determine spodumene quantification by using the matrix-matched standard calibration method. However, future work is still required to evaluate the feasibility of this proposed tof-SIMS quantification strategy for other different geological samples.

## Figures and Tables

**Table 1:** The normalized element sensitivities of spodumene 503R in tof-SIMS analysis.

*IS*	Element	Raster 1	Raster 2	Raster 3	Raster 4	Raster 5	Raster 6
Al	Li	16,783	19,333	19,588	20,068	19,732	19,535
	Na	406,209	467,922	474,098	485,705	477,575	472,809
	Al	6202	7144	7238	7416	7291	7219
	Si	1075	1238	1255	1285	1264	1251
	Mn	738,830	851,076	862,309	883,420	868,634	859,965
	Fe	62,219	71,671	72,617	74,395	73,150	72,420
Si	Li	16,583	19,504	19,680	20,117	19,861	19,732
	Na	401,346	472,049	476,306	486,891	480,706	477,566
	Al	6128	7207	7272	7434	7339	7291
	Si	1062	1249	1261	1289	1272	1264
	Mn	729,985	858,582	866,326	885,577	874,327	868,616
	Fe	61,474	72,303	72,955	74,577	73,629	73,148

**Table 2:** Element concentration results of spodumene 503R by tof-SIMS analysis ^1^.

*IS*	Element	Raster 1	Raster 2	Raster 3	Raster 4	Raster 5	Raster 6	Average	2σ	RSD%
Al	Li_2_O	7.89	6.97	7.60	7.79	7.60	7.70	7.59	0.24	4.3
	Na_2_O	0.094	0.085	0.089	0.091	0.087	0.088	0.089	0.002	3.5
	Al_2_O_3_	27.59	27.59	27.59	27.59	27.59	27.59	27.59	–	–
	SiO_2_	63.13	64.46	64.20	64.06	64.32	64.54	64.12	0.38	0.8
	MnO	0.058	0.054	0.060	0.057	0.059	0.058	0.058	0.001	3.4
	FeO	0.24	0.22	0.24	0.23	0.24	0.24	0.24	0.01	3.3
	Total	99.02	99.40	99.78	99.82	99.89	100.2	99.69	0.31	0.4
Si	Li_2_O	7.99	6.91	7.56	7.77	7.55	7.62	7.57	0.27	4.8
	Na_2_O	0.095	0.084	0.088	0.091	0.086	0.087	0.089	0.003	4.3
	Al_2_O_3_	27.93	27.35	27.47	27.53	27.42	27.32	27.50	0.17	0.8
	SiO_2_	63.90	63.90	63.90	63.90	63.90	63.90	63.90	–	–
	MnO	0.059	0.054	0.060	0.057	0.058	0.057	0.058	0.002	3.6
	FeO	0.25	0.22	0.24	0.23	0.23	0.24	0.23	0.007	3.8
	Total	100.2	98.53	99.32	99.57	99.24	99.22	99.35	0.41	0.6

^1^ The element concentration was given in oxide form and expressed as wt %.

**Table 3:** Comparison of spodumene 503R quantification by different analytical methods ^1^.

Method	No.	Li_2_O	Al_2_O_3_	SiO_2_	Na_2_O	MnO	FeO
tof-SIMS	Raster 1	7.97	27.87	63.76	0.095	0.058	0.25
	Raster 2	7.02	27.76	64.86	0.085	0.055	0.23
	Raster 3	7.61	27.66	64.34	0.089	0.061	0.24
	Raster 4	7.81	27.65	64.17	0.091	0.057	0.23
	Raster 5	7.61	27.62	64.39	0.087	0.059	0.24
	Raster 6	7.68	27.53	64.40	0.088	0.058	0.24
	Average	7.62	27.68	64.32	0.089	0.058	0.24
	2σ	0.27	0.10	0.29	0.003	0.002	0.007
LA-ICPMS	Spot 1	7.93	27.59	63.96	0.108	0.060	0.24
	Spot 2	8.03	27.82	63.71	0.113	0.053	0.20
	Spot 3	8.09	27.94	63.52	0.099	0.055	0.23
	Spot 4	8.04	27.64	63.70	0.101	0.066	0.25
	Spot 5	7.95	27.17	64.24	0.090	0.052	0.23
	Spot 6	8.16	27.63	63.77	0.081	0.048	0.23
	Spot 7	8.03	27.68	63.85	0.086	0.055	0.24
	Spot 8	7.98	27.71	63.88	0.077	0.046	0.22
	Spot 9	7.89	27.25	64.40	0.077	0.066	0.26
	Spot 10	7.96	27.53	63.93	0.074	0.064	0.24
	Average	8.00	27.59	63.90	0.091	0.057	0.23
	2σ	0.05	0.15	0.16	0.009	0.004	0.010
EPMA	Point 1	–	27.30	63.45	0.100	0.015	0.08
	Point 2	–	27.69	64.87	0.059	0.030	0.09
	Point 3	–	27.40	63.52	0.093	0.060	0.02
	Point 4	–	27.15	63.60	0.044	0.098	0.08
	Point 5	–	27.22	63.83	0.076	0.053	0.10
	Point 6	–	27.33	63.78	0.057	0.023	0.14
	Point 7	–	27.55	64.10	0.045	0.053	0.25
	Point 8	–	27.45	63.41	0.065	0.030	0.18
	Point 9	–	27.62	64.60	0.122	0.059	0.14
	Point 10	–	27.40	63.73	0.045	0.053	0.12
	Average	–	27.41	63.89	0.071	0.047	0.12
	2σ	–	0.10	0.30	0.016	0.018	0.038
*R*_tof-SIMS/LA-ICPMS_		0.95	1.00	1.01	0.98	1.02	1.01
*R*_tof-SIMS/EPMA_		–	1.01	1.01	1.26	1.22	1.99

^1^ The element concentration was given in oxide form and expressed as wt %.

**Table 4:** Element concentrations of spodumene 650 by the proposed tof-SIMS method ^1^.

Method		Li_2_O	Al_2_O_3_	SiO_2_	Na_2_O	MnO	FeO
tof-SIMS	Raster 1	7.93	26.92	64.87	0.115	0.145	0.0188
	Raster 2	7.94	27.41	64.36	0.117	0.149	0.0197
	Raster 3	8.00	27.38	64.33	0.118	0.148	0.0197
	Raster 4	7.50	26.53	65.69	0.108	0.153	0.0183
	Raster 5	7.81	27.42	64.48	0.115	0.149	0.0205
	Raster 6	7.85	27.67	64.19	0.117	0.150	0.0204
	Average	7.84	27.22	64.65	0.115	0.149	0.0196
	2σ	0.15	0.003	0.34	0.457	0.002	0.001
LA-ICPMS ^2^		7.92 ± 0.12	27.44 ± 0.18	64.33 ± 0.23	0.12 ± 0.004	0.15 ± 0.019	0.02 ± 0.007
EPMA ^2^		–	27.45 ± 0.12	64.5 ± 0.12	0.11 ± 0.014	0.12 ± 0.021	0.02 ± 0.012

^1^ The element concentration was given in oxide form and expressed as wt %; ^2^ The concentrations given by LA-ICPMS and EPPMA analysis were from our previous work [21].

## Data Availability

Data are contained within the article.

## Supplementary Materials

The following supporting information can be downloaded at: https://www.mdpi.com/article/10.3390/molecules30071552/s1, Figure S1: Mass spectra of spodumene under positive and negative ion mode; Figure S2: The BSE image of spodumene 503R; Figure S3: Mass spectra of NIST SRM 610 after different gas sputtering time; Figure S4: Mass spectra of spodumene 503R after different gas sputtering time; Table S1: Element concentration results of spodumene 650 by tof-SIMS analysis.

## References

[1] Macholdt D.S., Jochum K.P., Pöhlker C., Stoll B., Weis U., Weber B., Müller M., Kappl M., Buhre S., Kilcoyne A. (2015). Microanalytical methods for in-situ high-resolution analysis of rock varnish at the micrometer to nanometer scale. Chem. Geol..

[2] Li X.H., Li Q.L. (2016). Major advances in microbeam analytical techniques and their applications in Earth Science. Sci. Bull..

[3] Pownceby M.I., MacRae C.M., Wilson N.C. (2007). Mineral characterisation by EPMA mapping. Miner. Eng..

[4] Pisonero J., Fernández B., Günther D. (2009). Critical revision of GD-MS, LA-ICP-MS and SIMS as inorganic mass spectrometric techniques for direct solid analysis. J. Anal. At. Spectrom..

[5] Yang P., Wu G., Nuriel P., Nguyen A.D., Chen Y., Yang S., Feng Y., Ren Z., Zhao J. (2021). In situ LA-ICPMS Upb dating and geochemical characterization of fault-zone calcite in the central Tarim Basin, northwest China: Implications for fluid circulation and fault reactivation. Chem. Geol..

[6] Harris B.J.R., De Hoog J.C.M., Halama R. (2022). The behaviour of nitrogen during subduction of oceanic crust: Insights from in situ SIMS analyses of high-pressure rocks. Geochim. Cosmochim. Acta.

[7] Ma M., Wan Y., Santosh M., Xu Z., Xie H., Dong C., Liu D., Guo C. (2012). Decoding multiple tectonothermal events in zircons from single rock samples: SHRIMP zircon U–Pb data from the late Neoarchean rocks of Daqingshan, North China Craton. Gondwana Res..

[8] Massonnet P., Heeren R.M.A. (2019). A concise tutorial review of TOF-SIMS based molecular and cellular imaging. J. Anal. At. Spectrom..

[9] Stephan T. (2001). TOF-SIMS in cosmochemistry. Planet. Space Sci..

[10] Hagenhoff B. (2000). High resolution surface analysis by TOF-SIMS. Microchim. Acta.

[11] Long T., Clement S.W.J., Xie H., Liu D. (2020). Design, construction and performance of a TOF-SIMS for analysis of trace elements in geological materials. Int. J. Mass. Spectrom..

[12] Wickramasinghe R.C., Pasterski M.J., Kenig F., Ievlev A.V., Lorenz M., Gross J.M., Hanley L. (2021). Femtosecond laser desorption postionization ms vs tof-sims imaging for uncovering biomarkers buried in geological samples. Anal. Chem..

[13] North R., Tanner D., Nancarrow M., Pasic B., Mavrogenes J.A. (2022). Resolving sub-micrometer-scale zonation of trace elements in quartz using TOF-SIMS. Am. Mineral..

[14] North R., White L.T., Nancarrow M., Dosseto A. (2023). Sub-micrometre resolution FIB-SEM-based ToF-SIMS used to map geochemical zoning in four zircon reference materials. Geostand. Geoanal. Res..

[15] Saunders K., Rinnen S., Blundy J., Dohmen R., Klemme S., Arlinghaus H.F. (2012). TOF-SIMS and electron microprobe investigations of zoned magmatic orthopyroxenes: First results of trace and minor element analysis with implications for diffusion modeling. Am. Mineral..

[16] Song T., Liu J., Zhang C., Yang X., Chen T., Jiang S., Xu F., Li N., Zhu M., Li S. (2025). Characterization of the micro-morphology and compositional distribution of Chang’e-5 lunar soil mineral surfaces using TOF-SIMS. Ad. Sci..

[17] Marques A.F.A., Scott S.D., Sodhi R.N.S. (2011). Determining major and trace element compositions of exposed melt inclusions in minerals using ToF-SIMS. Surf. Interface Anal..

[18] Wang M.Q., Cai K.D., Li Z.P., Guo C. (2024). Quantitative accuracy assessment of trace elements and halogens in apatite by time-of-flight secondary ion mass spectrometry (TOF-SIMS). J. Anal. At. Spectrom..

[19] Graham J. (1975). Some notes on α-spodumene, LiAlSi_2_O_6_. Am. Mineral..

[20] Dessemond C., Lajoie-Leroux F., Soucy G., Laroche N., Magnan J.-F. (2019). Spodumene: The lithium market, resources and processes. Minerals.

[21] Tan X.J., Koch J., Günther D., Reusser E., Hattendorf B. (2021). In situ element analysis of spodumenes by fs-LA-ICPMS with non-matrix-matched calibration: Signal beat and accuracy. Chem. Geol..

[22] Liu C., Wang R.C., Wu F.Y., Xie L., Liu X.-C., Li X.-K., Yang L., Li X.-J. (2020). Spodumene pegmatites from the Pusila pluton in the higher Himalaya, South Tibet: Lithium mineralization in a highly fractionated leucogranite batholith. Lithos.

[23] Rinaldi R., Llovet X. (2015). Electron probe microanalysis: A review of the past, present, and future. Microsc. Microanal..

[24] Stevie F.A., Griffis D.P. (2008). Quantification in dynamic SIMS: Current status and future needs. Appl. Surf. Sci..

[25] Van der Heide P. (2014). Secondary Ion Mass Spectrometry: An Introduction to Principles and Practices.

[26] Longerich H.P., Jackson S.E., Gunther D. (1996). Laser ablation inductively coupled plasma mass spectrometric transient signal data acquisition and analyte concentration calculation. J. Anal. At. Spectrom..

[27] Werner H.W., Fiermans L., Vennik J., Dekeyser W. (1978). Introduction to Secondary Ion Mass Spectrometry (SIMS). Electron and Ion Spectroscopy of Solids.

[28] Jochum K.P., Nohl U., Herwig K., Lammel E., Stoll B., Hofmann A.W. (2005). GeoReM: A new geochemical database for reference materials and isotopic standards. Geostand. Geoanal. Res..

[29] Jochum K.P., Weis U., Stoll B., Kuzmin D., Yang Q., Raczek I., Jacob D.E., Stracke A., Birbaum K., Frick D.A. (2011). Determination of reference values for NIST SRM 610–617 glasses following ISO guidelines. Geostand. Geoanal. Res..

[30] Denny A.C., Zimmer M.M., Cunningham H.S., Sievers N.E. (2024). Sources of Li isotope bias during SIMS analysis of standard glasses. Chem. Geol..

[31] Ray G., Hart S.R. (1982). Quantitative analysis of silicates by ion microprobe. Int. J. Mass. Spectrom. Ion. Phys..

[32] Andersen C.A., Hinthorne J.R. (1972). Ion microprobe mass analyzer. Science.

[33] Tan X.J., Ren Y.X., Liang T., Wang D. (2025). Study of ultrasound-assisted low-pressure closed acid digestion method for trace element determination in rock samples by inductively coupled plasma mass spectrometry. Molecules.

[34] Xiong D., Guo L., Liu C., Wang L., Liu Y., Tan X. (2022). Analytical effect of stabilizer volume and shape on zircon U–Pb dating by nanosecond LA-ICP-QMS. J. Anal. Sci. Technol..

[35] Fricker M.B. (2012). Design of Ablation Cells for LA-ICP-MS. Ph.D. Thesis.

[36] Liu Y.S., Hu Z.C., Gao S., Gunther D., Xu J., Gao C.G., Chen H.H. (2008). In situ analysis of major and trace elements of anhydrous minerals by LA-ICP-MS without applying an internal standard. Chem. Geol..

[37] Armstrong J.T. (1995). CITZAF: A package of correction programs for the quantitative electron microbeam X-ray analysis of thick polished materials, thin Films, and particles. Microbeam Anal..

