# Supplementary material for: Quantitative Study of Spodumene by Time-of-Flight Secondary Ion Mass Spectrometry (tof-SIMS)
Source: Molecules. 2025 Mar 31;30(7):1552. doi: 10.3390/molecules30071552 (PMC11990334; doi:10.3390/molecules30071552)
Supplement: Supplementary file 1 [file molecules-30-01552-s001.zip › molecules-3524447-supplementary.pdf]

Article

Quantitative study of spodumene by time of flight secondary ion mass spectrometry (tof-SIMS)

Xijuan Tan [1]*

[1] Laboratory of Mineralization and Dynamics, College of Earth Sciences and Land Resources, Chang'an University, 126 Yanta Road, Xi'an, 710054, China;
* Correspondence: Xijuan Tan (tanxijuan@hotmail.com; tanxijuan@chd.edu.cn);

Supporting information

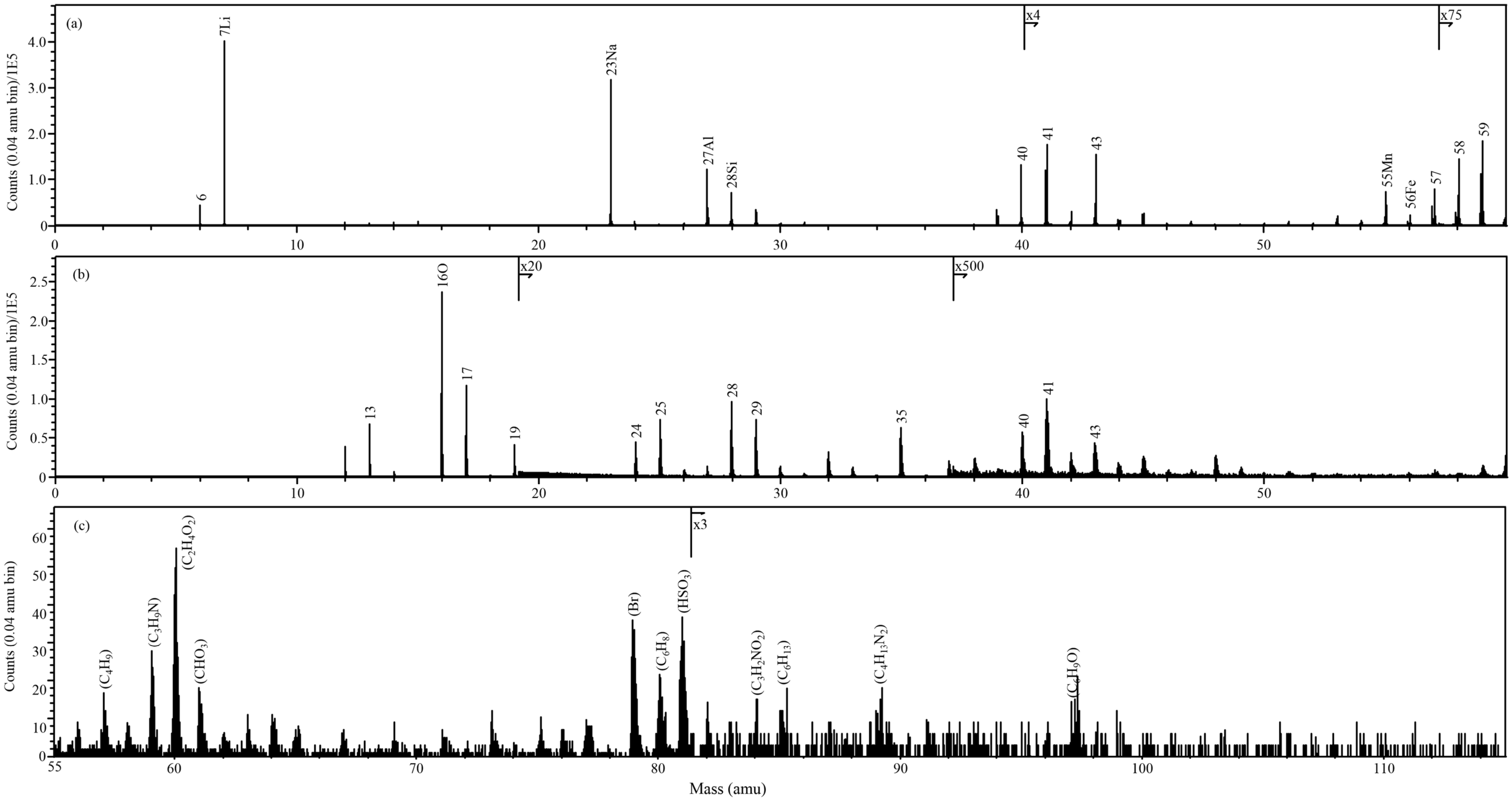

Figure S1. Mass spectras of spodumene under positive and negative ion mode. Here, the mass range was given from 0 to 60 amu. Pannel (a): positive ion mode, pannels (b) and (c): negative ion mode. The masses of Li, Na, Al, Si, Mn, Fe and O were shown as integer, which were 7, 23, 27, 28, 55, 56 and 16, respectively. Under negative ion mode, the signal intensities from 55 to 115 amu were less than hundred cts, with masses defaultly labelled by the software TOF-DR.

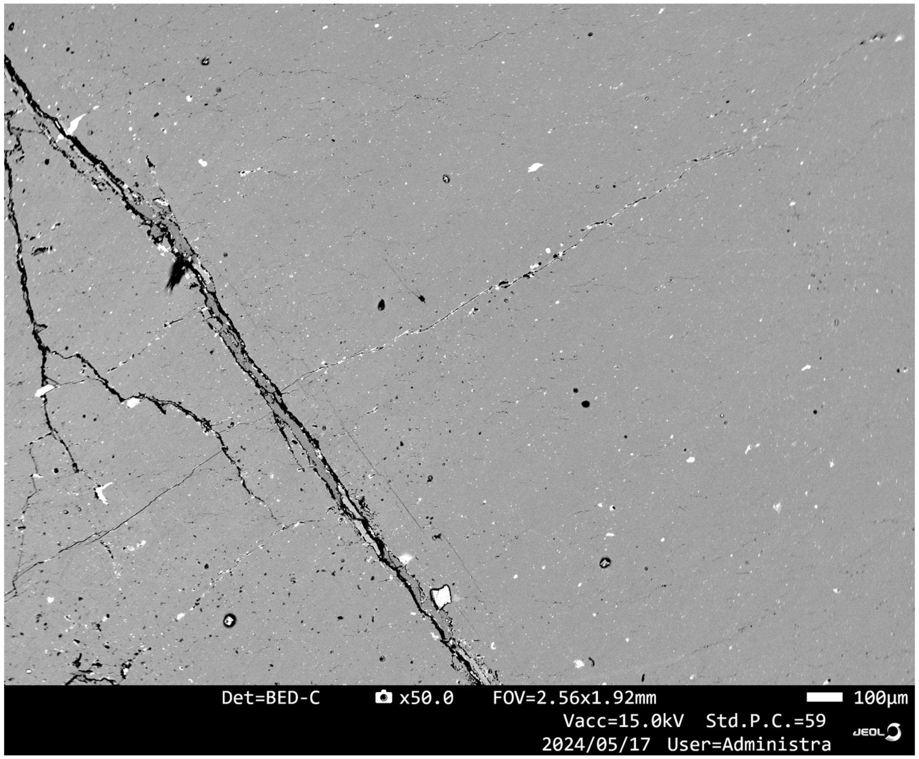

Figure S2. The BSE image of spodumene 503 R.

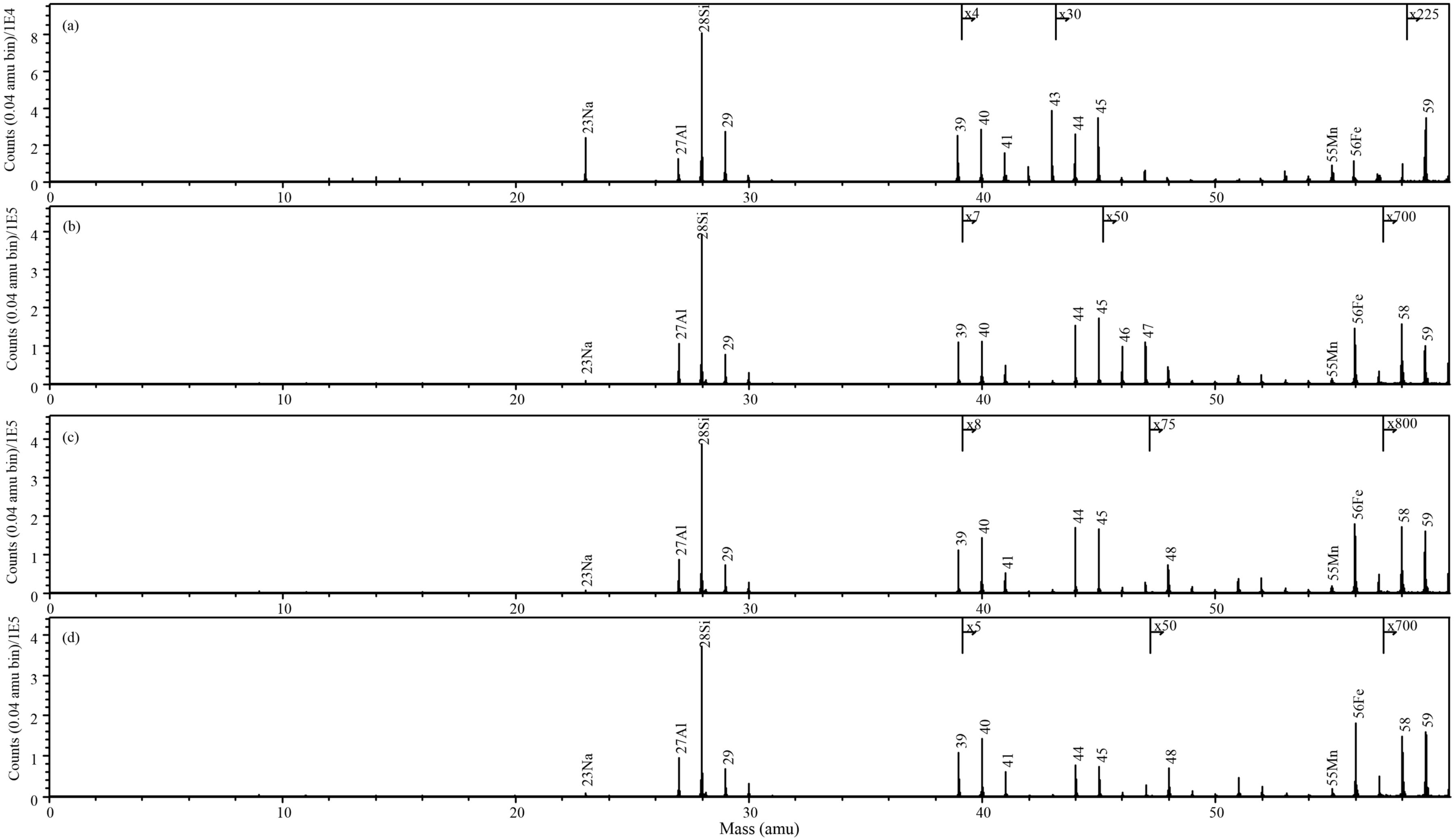

Figure S3. Mass spectra of NIST SRM 610 after different gas sputtering time. Here, the mass range was given from 0 to 60 amu. Pannels (a) to (d) were results with corresponding sputtering time of 0, 20, 40 and 60 s. The masses of Li, Na, Al, Si, Mn and Fe were shown as integer, which were 7, 23, 27, 28, 55 and 56, respectively. Only the studied elements were marked with chemical symbol, but Li was not shown due to extremly low intensity.

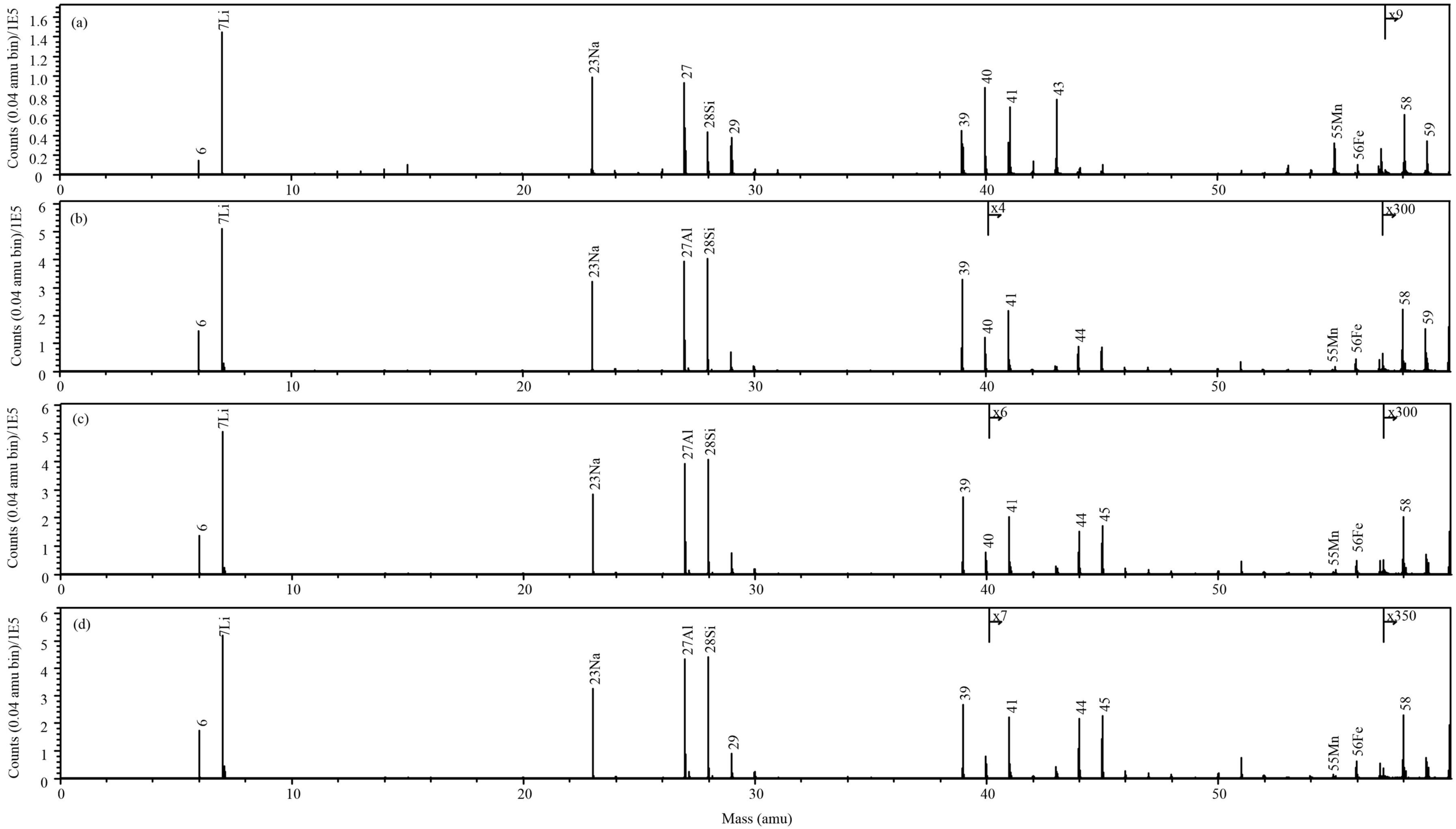

Figure S4. Mass spectra of spodumene 503R after different gas sputtering time. Here, the mass range was given from 0 to 60 amu. Pannels (a) to (d) were results with corresponding sputtering time of 0, 20, 40 and 60 s. The masses of Li, Na, Al, Si, Mn and Fe were shown as integer, which were 7, 23, 27, 28, 55 and 56, respectively. Only the studied elements were marked with chemical symbol.

Table S1. Element concentration results of spodumene 650 by tof-SIMS analysis [1].

IS	Element	Raster 1	Raster 2	Raster 3	Raster 4	Raster 5	Raster 6	Average	2σ	RSD%
Al	Li_2O	8.08	7.95	8.02	7.76	7.82	7.79	7.90	0.10	1.7
	Na_2O	0.12	0.12	0.12	0.11	0.12	0.12	0.12	0.002	2.0
	Al_2O_3	27.45	27.45	27.45	27.45	27.45	27.45	27.45	–	–
	SiO_2	66.14	64.44	64.49	67.98	64.54	63.68	65.21	1.174	2.4
	MnO	0.15	0.15	0.15	0.16	0.15	0.15	0.15	0.003	2.6
	FeO	0.0192	0.0198	0.0198	0.0189	0.0206	0.0202	0.0197	0.0005	3.1
	Total	102.0	100.1	100.3	103.5	100.1	99.21	100.9	1.17	1.6
Si	Li_2O	7.88	7.96	8.02	7.36	7.82	7.89	7.82	0.18	3.0
	Na_2O	0.11	0.12	0.12	0.11	0.12	0.12	0.12	0.003	3.9
	Al_2O_3	26.77	27.47	27.45	26.05	27.44	27.80	27.16	0.4791	2.4
	SiO_2	64.50	64.50	64.50	64.50	64.50	64.50	64.50	–	–
	MnO	0.14	0.15	0.15	0.15	0.15	0.15	0.15	0.002	1.5
	FeO	0.0187	0.0198	0.0198	0.0180	0.0205	0.0205	0.0195	0.0008	5.2
	Total	99.43	100.2	100.3	98.18	100.0	100.5	99.77	0.64	0.9

[1] The element concentration was given in oxide form and expressed as wt %.
